# Photopatterning of organic mixed ionic electronic conductors for monolithic complementary inverter

**DOI:** 10.1016/j.isci.2026.114965

**Published:** 2026-02-09

**Authors:** Xinyao Xie, Linlong Zhang, Mingyu Ma, Gang Ye, Jun Zhang, Xingxing Chen, Minghui You, Jian Liu

**Affiliations:** 1 College of Information Technology, Jilin Agricultural University, Changchun 130000, P.R. China; 2 State Key Laboratory of Polymer Science and Technology, Changchun Institute of Applied Chemistry, Chinese Academy of Sciences, Changchun 130022, P.R. China; 3 School of Applied Chemistry and Engineering, University of Science and Technology of China, Hefei 230026, P.R. China; 4 College of Engineering, Changchun Normal University, Changchun 130022, P.R. China; 5 School of Chemistry and Chemical Engineering, Anhui University, Hefei, Anhui 230601, P.R. China

**Keywords:** Applied sciences, Circuit systems, Engineering

## Abstract

High-resolution semiconductor patterning is critical for integrated organic complementary logic circuits. We report a photopatterning strategy based on the rational blending of conjugated polymers and photo-crosslinkable small molecules. The systematic optimization of crosslinker ratio reveals a critical window that yields 151% enhancement in charge carrier mobility for the n-type organic mixed ionic electronic conductor (OMIEC). The crosslinked films achieve enhanced charge carrier mobility (*μ* = 0.212 cm^2^ V^−1^ s^−1^) by balancing ion penetration and charge carrier transport through the formation of tailored, dense crosslinking networks. The films exhibit exceptional solvent resistance for over 1 h, enabling sequential multilayer circuit integration without interfacial degradation. This optimized system enables the fabrication of a cofacially integrated organic complementary inverter with a peak gain of 221 VV^-1^. This work provides mechanistic insight into photo-crosslinkable OMIECs optimization and a practical route to monolithic organic circuits.

## Highlights

• A photopatterning strategy enables high-resolution patterning of OMIECs
• Systematic optimization of crosslinker ratio boosts charge carrier mobility (*μ*) by 151%
• The OMIEC patterns show robust solvent resistance for multilayer circuit integration
• Monolithically integrated complementary inverter demonstrates a high gain of 221 VV^-1^

## Graphical abstract

**Figure:**
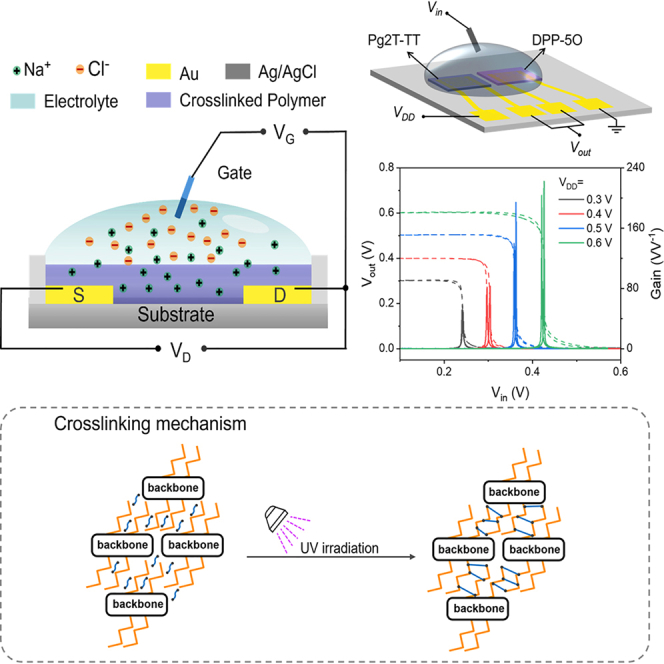

## Introduction

Organic electrochemical transistors (OECTs) are ion-to-electron transducers that utilize organic semiconductor channels for signal transduction.^1^^,^^2^^,^^3^ These devices operate via electrochemical doping, where electrolyte ions permeate the channel to compensate for the charges injected by the source or drain electrode, enabling the electrolyte-gated modulation of the source-drain current through ion injection.^4^^,^^5^^,^^6^^,^^7^^,^^8^^,^^9^ These specific organic semiconductors are organic mixed ionic-electronic conductors (OMIECs), which offer advantages such as good biocompatibility, low-cost solution-processability, high transconductance, low operating voltage, and efficient ion-electron coupling.^10^^,^^11^^,^^12^^,^^13^^,^^14^ These features make OECTs highly suitable for biosensing applications, such as biomarker detection and recording of electrophysiological signals.^15^^,^^16^^,^^17^^,^^18^ Matching p-type and n-type OECTs enables the construction of an organic complementary inverter capable of signal processing and amplification, which is key to integrating organic electronics into bioelectronic systems.^19^^,^^20^^,^^21^^,^^22^ The integrated OECT-based biosensors enable intelligent medical devices capable of real-time signal processing and adaptive response.^23^^,^^24^^,^^25^ OECT-based organic circuits show great potential for monolithic bioelectronic systems where ion-mediated switching enables direct interface between biochemical inputs and electronic processing.^26^^,^^27^^,^^28^

Complementary inverters are implemented by electrically connecting p-type and n-type OECTs in series, with matching threshold voltages, transconductance, and response times.^2^^,^^18^^,^^29^ Integrating p-type and n-type OECTs on a cofacial substrate to construct monolithic complementary inverters can significantly enhance circuit switching speed, simplify circuit design, and advance the development of an organic complementary inverter with a low operating voltage. Such a cofacial complementary inverter can be fabricated from a single ambipolar polymer using low-cost methods, such as spin-casting a polymer solution. However, the characteristics of single ambipolar materials exhibit a certain level of conductivity when transporting both holes and electrons, which prevents the inverter from fully switching off in the “0” and “1” states.^30^^,^^31^ High-resolution patterning enables the miniaturization of cofacial complementary inverters from unipolar OECTs, thereby facilitating the development of small, energy-efficient organic electronic devices.^32^^,^^33^ Effective patterning of polymer semiconductors is a key step in fabricating integrated electronic devices based on unipolar OECTs, thereby reducing source-drain leakage current, crosstalk, and power consumption between adjacent devices.^34^^,^^35^ To date, some methods for patterning polymer semiconductors have been reported. For example, solution-processing techniques, such as using polydimethylsiloxane (PDMS), can achieve precise patterning via solution-processed thin-film growth.^36^ Physical vapor transport methods enable the selective growth of patterned arrays of organic semiconductors on patterned substrates.^37^ Inkjet printing deposits organic semiconductor materials at predefined locations.^36^^,^^38^ Nanoimprint lithography is also a simple method for fabricating organic semiconductor arrays.^39^^,^^40^ Although these patterned arrays of organic semiconductors exhibit promising semiconductor properties, the practical application of these semiconductor patterns in all solution-processed electronic devices remains a challenge. This is attributed to the fact that solvents used for processing other layers may not be orthogonal to the organic semiconductor, leading to irreversible damage.^41^^,^^42^^,^^43^ In addition to these, conventional photoresists (SU-8 and AZ series) can achieve high-resolution patterning of individual p-type or n-type semiconductors, characterized by their high aspect ratios. However, during the fabrication of multiple-layer complementary logic circuits, pre-deposited semiconductor layers are vulnerable to degradation when exposed to photoresist solvents, developers, and stripping solutions. Chemical exposure often leads to film damage and irreversible deterioration of the mixed-conduction performance of semiconductors, ultimately compromising the functionality of the resulting complementary logic circuits. Recent studies have effectively demonstrated the photo-crosslinker DtFDA for patterning both p-type and n-type OMIECs individually, achieving high-resolution features and functional organic electrochemical transistors (OECTs) and inverters.^19^^,^^44^ These reports underscore the general applicability of DtFDA. However, a systematic investigation into how the mass ratio of crosslinker to polymer fundamentally influences the film’s microstructure, electrochemical properties, and ultimately the electronic transport performance remains underexplored, particularly for n-type materials.

In this work, we developed a universal and straightforward photo-crosslinking strategy for patterning OMIECs by blending the photo-crosslinkable small molecule DtFDA with the conjugated polymer DPP-5O. By optimizing the solvent and blending ratio, we achieved high-resolution photo-patterning of OMIECs with a high aspect ratio. Using an appropriate photomask, high-resolution regular patterns with a minimum line width/gap of 20 μm were conveniently prepared in the polymer film after 10 s of UV exposure and fast developing. The photo-patterned films were used to fabricate OECTs, and the charge carrier mobility (*μ*) of the devices achieved 151% of that of the corresponding pristine films. The crosslinked three-dimensional network restricts the movement of polymer chains through covalent bonding, suppressing the packing disorder induced by harmful swelling and promoting the stabilization and delocalization of polarons, thus enhancing charge transport.^45^^,^^46^^,^^47^ Moreover, the crosslinked films demonstrated exceptional resistance to polar solvents, including chloroform and hexafluoroisopropanol (HFIP), enabling successful multilayer deposition without interfacial degradation. Through sequential photolithographic patterning of n-type (DPP-5O) and p-type (Pg2T-TT) layers, we fabricated cofacial complementary inverters exhibiting a voltage gain of 221 VV^-1^ at a working voltage V_DD_ = 0.6 V. The maximum power consumption is lower than 0.12 μW at V_DD_ = 0.3 V. The photo-patterning strategy through optimizing crosslinker weight ratio successfully reconciles the requirements of high-resolution semiconductor patterning and high mixed ionic-electronic conduction performance, thereby realizing a monolithic organic complementary inverter based on sequential multilayer solution deposition.

## Results

### Preparation and characterization of polymer patterns

**Figure 1:**
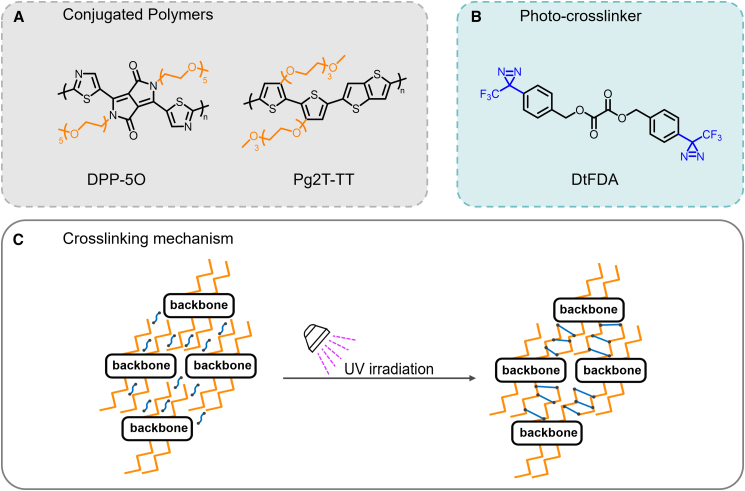
Materials and crosslinking mechanism Chemical structures of (A) conjugated polymers DPP-5O and Pg2T-TT and (B) photo-crosslinker molecular DtFDA. (C) The crosslinking mechanism of conjugated polymers and DtFDA.

**Figure 2:**
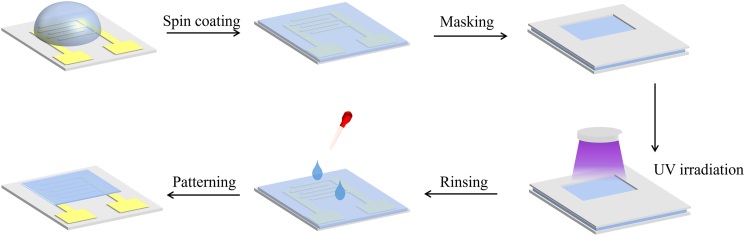
Photo-patterning process of the active layer of OECTs, including spin coating blends, masking for patterns, UV irradiation for photo-crosslinking, rinsing with developer, and the pattern

**Figure 3:**
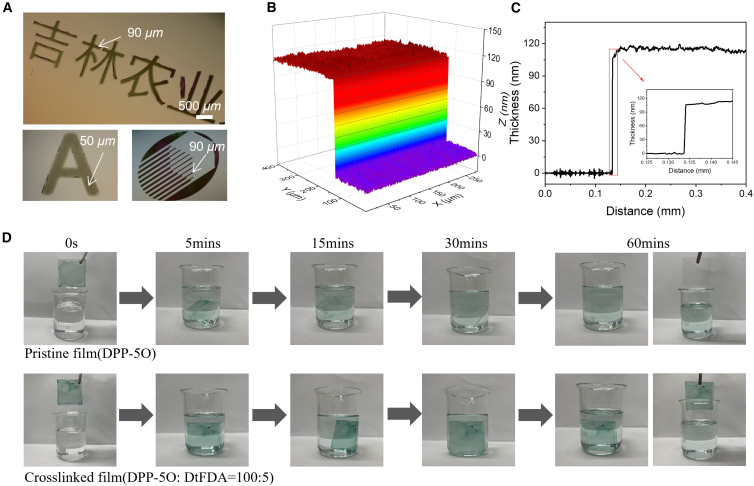
Optical images and solvent resistance of OMIEC patterns (A) Microscopic images of CF-processed patterns of text characters and irregular shapes. (B) Optical profilometry image and (C) the corresponding line-cuts curve of CF-processed photo-pattern based on crosslinked DPP-5O (DtFDA:DPP-5O = 10:100). (D) The crosslinked films were soaked in chloroform for different times.

Figures 1A and 1B displays the chemical structures of conjugated polymers DPP-5O and Pg2T-TT, and photo-crosslinker DtFDA. DtFDA is a small-molecule compound used for photo-crosslinking, featuring a diazirine structure with dual-end trifluoromethyl substitution.^44^ It has been studied and applied in organic electrochemical transistors (OECTs) and other organic electronic devices, primarily as a photo-crosslinker to achieve precise patterning and regulate ion transport.^44^^,^^48^ Since DtFDA crosslinks with semiconductors under UV light (the crosslinking mechanism are shown in Figure 1C) and exhibits good resistance to organic solvents, we blended it with polymers to form high-resolution, precise patterns. DPP-5O was dissolved in CF and HFIP (5 mg/mL) and mixed with DtFDA at a weight ratio of 100:10 to determine the optimized solvent. Figure 2 shows the photo-patterning process of the OMIEC blends. Figure 3A illustrates the microscopic images of CF-processed patterns of text characters and irregular shapes with a minimum line width/gap of 20 μm, exhibiting more precise patterning than the HFIP-processed counterpart (Figure S2). The optical profilometry image shows that the CF-processed film exhibited a uniform surface morphology and a distinct edge, as shown in Figure 3B. The lateral sidewall deviation of the OMIEC photo-patterning is 0.8 μm, which was extracted from the optical profilometry line-cuts curve (Figure 3C). The solvent resistance of the crosslinked films was then evaluated. Notably, the patterns remained completely intact, with no observable feature degradation, after soaking in chloroform for 60 min (Figure 3D). This exceptional solvent resistance enables the sequential solution-processing of multilayer device architectures without damaging underlying layers.

### Electrochemical doping of DPP-5O films

**Figure 4:**
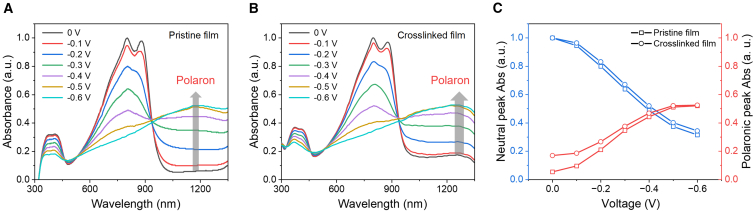
Spectroelectrochemistry of pristine DPP-5O and crosslinked DPP-5O The spectroelectrochemistry of thin films of (A) pristine DPP-5O and (B) crosslinked DPP-5O. (C) The plot of the total difference spectra of the intensities of neutral and polaronic absorption peaks of pristine DPP-5O and crosslinked DPP-5O.

Through photo-crosslinking lithography, high-resolution, solvent-resistant polymer patterning was achieved. Subsequently, spectroelectrochemical characterization was conducted to investigate the electrochemical properties of the polymer films. As illustrated in Figure S3, the UV-vis-NIR absorption spectra of DtFDA and DPP-5O blends in dilute solution exhibit identical absorption profiles across all tested weight ratios, with maximum absorption peaks at 776 and 854 nm, respectively. This indicates a lack of significant ground-state electronic interaction and pre-aggregation between the polymer and the crosslinker in solution. Then, the evolutions of absorption spectra with doping voltage of thin films of pristine DPP-5O and crosslinked DPP-5O (10 wt %) were investigated, as shown in Figures 4A and 4B. When applying no bias voltage, the two maximum neutral absorption peaks are 804 and 882 nm for the pristine DPP-5O film, indicating a bathochromic shift of 28 nm relative to the solution state. The crosslinked DPP-5O (10 wt %) film shows a red shift of 26 nm of two maximum neutral absorption peaks to 802 and 880 nm, which suggests a negligible difference in π-π∗ absorption characteristic in comparison with the pristine film. The results suggest that blending with DtFDA has little impact on the highly planar backbone and pre-aggregation of DPP-5O. Upon applying stepwise voltage from 0 to −0.6 V (step = 0.1 V), the intensities of these neutral peaks progressively attenuated, accompanied by the emergence of a polaronic absorption peak at 1177 nm for the pristine film and 1246 nm for the crosslinked film, indicating successful electrochemical doping within the polymer matrix.^49^^,^^50^^,^^51^ The bathochromic shift of polaronic peaks for the crosslinked film indicates enhanced stabilization and delocalization of the generated polarons. This can be attributed to the stabilization of polymer backbones and side chains induced by the formation of a robust cross-linked network, which effectively suppresses detrimental swelling-induced packing disruptions during ion injection. The enhanced polaron stabilization and delocalization were expected to reduce the energy barrier to charge-carrier transport in the polymer backbone, thereby improving the carrier mobility of OMIEC. Figure 4C shows the evolution of the total difference spectra of neutral and polaronic absorption characteristics of pristine and crosslinked films. The crosslinked film exhibits higher polaron absorption intensities at low electrochemical doping voltages than the pristine film. At the same time, both achieve similar neutral and polaronic absorption intensities at high doping levels (−0.6 V). It indicates that the crosslinked film can be doped more readily, facilitating the formation of charge carriers at lower driving forces. The spectroelectrochemical analysis reveals that the semiconductor photo-crosslinking strategy provides a robust, mixed-conductive network, thereby facilitating both optimized polaron delocalization and stabilization, as well as a more efficient electrochemical doping process.

### Organic electrochemical transistor devices performance

**Figure 5:**
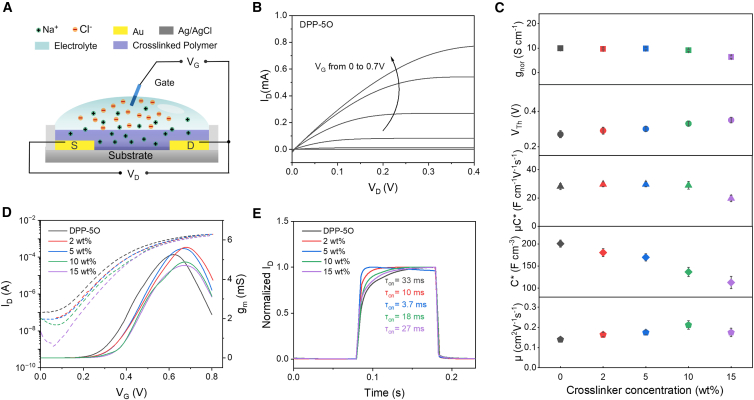
OECTs based on DPP-5O with varying crosslinker weight ratios (A) The device structure of OECT. (B) Output characteristics of pristine DPP-5O-based OECT. (C) OECT performance summary of DPP-5Os: normalized transconductance (gnor), threshold voltage (V_Th_), figure-of-merit (*μ*C∗), volumetric capacitance (C∗), and charge carrier mobility (*μ*). (D) Transfer characteristics and (E) transient behaviors of OECTs based on DPP-5Os.

In this section, we set out to fabricate the OECT as above, “device fabrication,” and measure the OECT performance of crosslinked DPP-5O with different ratios of DtFDA, as shown in Figures 5A–5E. The OECT device performance measurements were operated in an aqueous electrolyte (0.1 M NaCl), and an Ag/AgCl wire was used as the gate. When a gate voltage (V_G_) is applied, counterions in the electrolyte permeate the channel layer and compensate for electrons injected from the source electrode. Figures 5B and S4 show the output characteristics of DPP-5O-based OECTs, which exhibit a typical accumulation mode for crosslinked DPP-5O with varying DtFDA ratios. The output characteristics of DPP-5O-based OECTs exhibit linear and saturated regimes depending on the magnitude of V_D_. The transfer curves were measured in the saturated regime, as shown in Figure 5D. The OECTs based on crosslinked DPP-5O exhibited a significantly lower off-state current (∼10^−9^ A) compared to those based on the pristine polymer (∼10^−7^ A), representing a reduction of two orders of magnitude. The lower off-state current can be attributed to reduced leakage current resulting from denser crosslinking. The steady-state and transient behaviors of DPP-5O-based OECT devices were studied from transfer characteristics and I_D_-time response curves. Figures 4C–4E show the transient behaviors and values of g_nor_, V_Th_, *μ*C∗, C∗, and *μ* of DPP-5O films with crosslinker weight ratios of 0 (pristine DPP-5O), 2, 5, 10, and 15 wt %, respectively. The key OECT performance parameters of DPP-5Os with varying crosslinker weight ratios are summarized in Table 1. The g_nor_ of DPP-5Os were 9.9 ± 1.1, 9.7 ± 0.4, 9.8 ± 0.6, 9.1 ± 0.4, and 6.3 ± 0.5 S cm^−1^ for crosslinker weight ratios of 0, 2, 5, 10, and 15 wt %, respectively. The *μ*C∗ values of DPP-5Os were 28.1 ± 2.2, 29.6 ± 1.7, 29.7 ± 1.8, 28.9 ± 2.7, and 19.7 ± 2.3 F cm^−1^ V^−1^ s^−1^ for crosslinker weight ratios of 0, 2, 5, 10, and 15 wt %, respectively. The crosslinked DPP-5O-based devices could maintain high n-type performance with a low crosslinker ratio. Significant degradation of g_nor_ and *μ*C∗ was observed at a high weight ratio of 15 wt %, and should be attributed to an overly dense crosslinking network that hindered ion transport and disrupted the percolation pathways for electronic charge carriers. The volumetric capacitance (C∗) was extracted by analyzing electrochemical impedance spectroscopy (EIS) data using an equivalent circuit (Figures S5–S7). The C∗ of DPP-5Os were 200.8 ± 8.1, 180.5 ± 9.2, 169.8 ± 8.2, 136.6 ± 10.6, and 112.9 ± 13.9 F cm^−3^ for crosslinker weight ratios of 0, 2, 5, 10, and 15 wt %, respectively. It clearly shows that C∗ values decrease with increasing crosslinking degree, indicating that a higher gate voltage is required to drive enough counterions into the organic films and switch on the devices. Therefore, the crosslinked DPP-5O-based devices showed an increase in threshold voltage with increasing crosslinking degree. The mobility (*μ*) values were calculated from *μ*C∗ and C∗ of DPP-5O-based devices. The crosslinked DPP-5O-based devices exhibited higher mobility values, 0.164, 0.175, 0.212, and 0.175 cm^2^ V^−1^ s^−1^ for crosslinker weight ratios of 2, 5, 10, and 15 wt %, respectively, compared to 0.140 cm^2^ V^−1^ s^−1^ for the pristine DPP-5O-based device. Through a semiconductor crosslinking strategy, higher mobility was achieved, attributed to enhanced polaron stabilization and delocalization, as well as a lowered energy barrier for charge-carrier transport in the polymer backbone, as evidenced by spectroelectrochemical analysis. This intrinsic advantage also promoted device switching and achieved a faster transient response, with a switch-on time (τ_on_) of 18 ms for crosslinked DPP-5O (10 wt %) compared to 33 ms for pristine DPP-5O. The results indicate that the crosslinked network in DPP-5O (10 wt %) achieves a critical balance: it is robust enough to mitigate swelling-induced disorder, thus boosting mobility and switching speed, yet remains open enough to permit rapid ion penetration for efficient doping.

**Table 1:** Summary of key OECT performance parameters for DPP-5Os with varying crosslinker weight ratios

Crosslinker weight ratio	g_nor_^a^ (S cm^−1^)	V_Th_^b^ (V)	*μ*C∗^c^ (F cm^−1^ V^−1^ s^−1^)	τ_on_/τ_off_ (ms)	C∗^d^ (F cm^−3^)	*μ*^e^ (cm^2^ V^−1^ s^−1^)
0 (pristine)	9.9 ± 1.1	0.27	28.1 ± 2.2	33/1.3	200.8 ± 8.1	0.140 ± 0.012
2 wt %	9.7 ± 0.4	0.29	29.6 ± 1.7	10/1.0	180.5 ± 9.2	0.164 ± 0.010
5 wt %	9.8 ± 0.6	0.30	29.7 ± 1.8	3.7/1.0	169.8 ± 8.2	0.175 ± 0.012
10 wt %	9.1 ± 0.4	0.33	28.9 ± 2.7	18/1.3	136.6 ± 10.6	0.212 ± 0.023
15 wt %	6.3 ± 0.5	0.35	19.7 ± 2.3	27/1.4	112.9 ± 13.9	0.175 ± 0.021

a The transconductance normalized by the channel geometry factors.

b Determined by extrapolating the linear regime of the I_D_^1/2^-V_G_ plot.

c Extracted from the slope of g_m_ as a function of (Wd/L)(V_Th_-V_G_).

d Obtained by linear fitting of capacitance and volumes of channel materials.

e Calculated from the *μ*C∗ and the volumetric capacitance C∗.

Beyond initial electrical performance, operational stability in aqueous and physiological environments is crucial for practical OMIEC devices. To this end, we evaluated the cycling stability of the crosslinked films (Figure S8). In 0.1 M NaCl, a clear positive correlation was observed between the DtFDA crosslinker content and stability: films with higher crosslinker ratios exhibited significantly enhanced retention of their electrical characteristics over tens of cycles. This is attributed to the robust, three-dimensional covalent network that effectively confines the polymer chains, preventing excessive swelling, irreversible morphological changes, or material dissolution during redox cycling. Furthermore, the optimal film (15% DtFDA) showed equally outstanding stability in phosphate-buffered saline (0.01 M PBS, pH 7.4), a standard medium that mimics biological fluids, as displayed in Figure S9. This confirms that the stability enhancement conferred by crosslinking is effective not only in simple electrolytes but also in more complex, biologically relevant environments, underscoring the potential of this photopatterning strategy for future bioelectronic applications.

**Figure 6:**
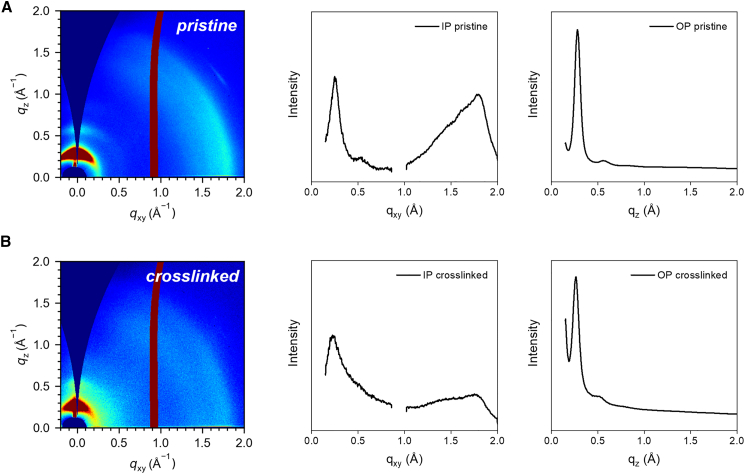
GIWAXS characterization of pristine and crosslinked DPP-5O films 2D GIWAXS patterns of (A) pristine and (B) crosslinked (10 wt %) DPP-5O films, and the corresponding in-plane and out-of-plane line cuts.

To gain insight into the microstructure supporting the enhanced electrical performance, we conducted two-dimensional (2D) grazing-incidence wide-angle X-ray scattering (GIWAXS) measurements on both pristine and crosslinked (10 wt %) DPP-5O films. As shown in Figures 6A and 6B, both films exhibited a predominant “edge-on” molecular orientation relative to the substrate, which is favorable for in-plane charge transport in OECTs. Quantitative analysis of the diffraction patterns revealed that the π-π stacking distance (d) slightly increased from 3.49 Å in the pristine film to 3.59 Å in the crosslinked film. The lamellar packing distances increased in-plane (IP) from 25.4 Å to 27.9 Å and out-of-plane (OP) from 22.4 Å to 23.7 Å after crosslinking. These increases in packing distances are attributed to the incorporation of the crosslinker DtFDA, which introduces a degree of structural expansion within the otherwise well-ordered polymer matrix. Crucially, the preservation of the sharp “edge-on” orientation and the minimal perturbation to the π-π stacking distance, which remains well within the range for efficient charge transport, explain the retained high mobility. Furthermore, the expanded lamellar spacing likely facilitates ion penetration and hydration in aqueous electrolytes, contributing to efficient electrochemical doping and the remarkable device performance.

### Applications of photo-patterned thin films

**Figure 7:**
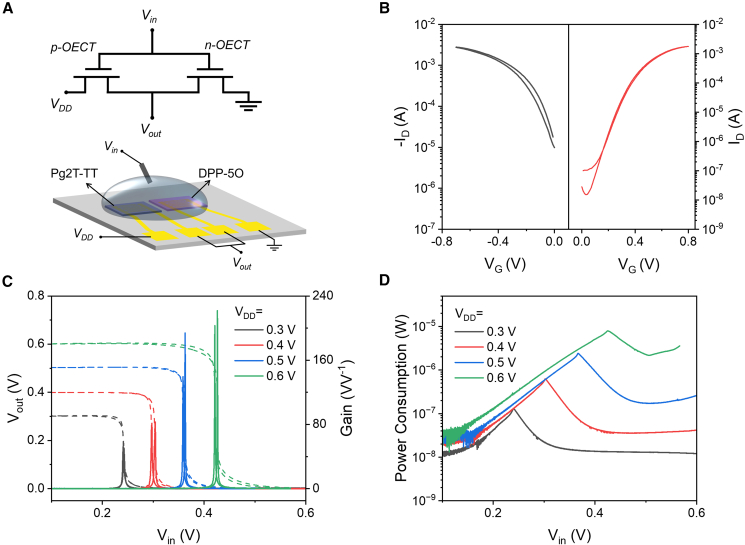
Cofacial complementary inverter based on the monolithic integration of p-type and n-type OECT (A) Circuit configuration and schematic diagram of the cofacial complementary inverter. P-type OECT: Pg2T-TT, and n-type OECT: DPP-5O. (B) Transfer curves of a single OECT device based on Pg2T-TT and DPP-5O. (C) Optimized voltage transfer characteristic and calculated voltage gain, and (D) power consumption curves of the cofacial complementary inverter.

In this section, we set out to fabricate the cofacial complementary inverter to achieve monolithic integration of p-type and n-type OECT. As shown in the circuit diagram Figure 7A, the source of the p-type OECT is connected to the supply working voltage (V_DD_), while the source of the n-type OECT is grounded. The two OECTs share a common Ag/AgCl electrode, which serves as the standard input voltage (V_in_). The drains of the two OECTs are externally connected to measure the output voltage (V_out)_. As shown in Figure 7B, the transfer characteristics of Pg2T-TT and DPP-5O exhibited balanced ambipolar charge transport and clear switching characteristics under gate voltage. The matched performance of the p-type and n-type channels is expected to enable a high voltage gain for the inverter.^2^^,^^31^ The voltage transfer characteristics (VTCs) and the corresponding voltage gains of the cofacial complementary inverter were recorded under varying V_DD_ (0.3, 0.4, 0.5, and 0.6 V) while sweeping V_in_ from 0 to 0.5 V, as displayed in Figures 7C and S10. As V_in_ gradually increases, the inverter fully switches from “1” to “0” states. The sharp V_out_ transition of the full rail-to-rail swing V_in_ from 0 to 0.6 V is enabled by low off-current and large sub-threshold swing of both p-type and n-type OECTs. The inverter gain was extracted from the VTC (∂V_out_/∂V_in_) and was found to depend on V_DD_. The voltage gains and maximum power consumption values of the cofacial complementary inverter are 52, 83, 194, and 221 VV^-1^, and 0.12, 0.62, 2.4, and 7.8 μW, respectively, at V_DD_ of 0.3, 0.4, 0.5, and 0.6 V (Figure 7D).

### Comparative analysis of the photo-patterning strategy

As previously mentioned, techniques such as e-beam lithography, nanoimprint lithography, conventional lithography, and inkjet printing are capable of patterning organic semiconductors. A comparison in terms of resolution, process throughput, and material compatibility of these patterning techniques for OMIECs is displayed in Table S1. Although e-beam lithography achieves outstanding resolution and nanoimprint lithography offers moderate nanoscale throughput, their reliance on complex, subtractive, or physically invasive processes often restricts compatibility with sensitive OMIECs and sequential solution-based multilayer integration. In comparison, the photo-crosslinking approach presented here balances micron-scale resolution with an extremely simple and high-throughput process. Crucially, it not only prevents performance degradation but also actively enhances OMIEC properties and enables robust multilayer stacking by imparting solvent resistance. This addresses key integration challenges encountered with both conventional lithography and direct-write techniques such as inkjet printing.

## Discussion

In this study, we developed a novel OMIEC photo-patterning strategy based on the blend of conjugated polymers and photo-crosslinkable small molecules. Efficient crosslinking was achieved with a short exposure time of 10 s and a low additive content (<10 wt %), enabling high-resolution photo-patterning of organic mixed ionic-electronic conductors (OMIECs). The crosslinked patterned films exhibited high mixed ionic-electronic conduction, with carrier mobility 151% that of pristine films. The crosslinked OECT devices exhibited good uniformity in performance, and the films showed excellent solvent resistance while maintaining large current on/off ratios and high transconductance. We successfully fabricated cofacial complementary inverters with fully independent p-type and n-type channels on a single substrate using a two-step double-patterning process. These devices achieved high gain at a low working voltage, highlighting the practical application potential of this polymer blend and crosslinker system in advanced organic electronics. In summary, this work presents a strategy for developing efficient device fabrication processes for organic semiconductors. It facilitates the preparation of multilayer electronic devices with superior UV crosslinking and high-resolution patterning, demonstrating great potential in multilayer organic electronics.

The photolithographic patterning demonstrated in this work achieves a feature size of 20 μm, which is suitable for numerous applications in flexible microelectronics, such as biosensor arrays and large-area interactive skins. However, for the future development of high-density organic integrated circuits, advancing the resolution toward the sub-micrometer scale remains a key challenge. Future work can follow a clear roadmap to exploit the patterning compatibility of this material system. First, the photochemical process could be refined by using advanced lithographic techniques such as two-photon absorption lithography or by optimizing the development conditions to reduce line edge roughness. Additionally, material engineering holds great potential, including the design of photo-crosslinkers with higher sensitivity and smaller molecular footprints. Furthermore, exploring optical methods such as using light sources with shorter wavelengths (deep-UV) or implementing near-field lithography setups could directly overcome the diffraction limit associated with the current photomask-based process. Pursuing these directions will be essential to push the boundaries of our technology toward the demands of next-generation, ultra-dense organic electronic devices.

### Limitations of the study

While the photo-patterning strategy presented here offers a promising foundation, its translation into practical implantable bioelectronic systems faces two principal challenges: first, the long-term operational stability under dynamic biological environments requires further validation; and second, the reliable integration of soft, hydrated semiconductor patterns with rigid device components needs to be developed. Addressing these challenges represents an essential next step toward advancing from a patterned film to a fully functional, chronically implantable device.

## Resource availability

### Lead contact

Further information and requests for resources should be directed to and will be fulfilled by the lead contact Jian Liu (jian.liu@ciac.ac.cn).

### Materials availability

This study did not generate new unique reagents.

### Data and code availability

• Date reported in this article will be shared by the lead contact upon request.
• This article does not report original code.
• Any additional information required to reanalyze the data reported in this article is available from the lead contact upon request.

## Acknowledgments

The project is supported by the 10.13039/501100011789Jilin Provincial Department of Science and Technology, No. 20240402057GH, and the National Natural Science Foundation of China (Nos. 62204095, 61774025), the 10.13039/501100003512State Administration of Foreign Experts Affairs (No. D20240079, D20240241, the 10.13039/501100003995Anhui Provincial Natural Science Foundation (2408085Y028), and the National Natural Science Foundation of China (22205004).

We thank the 1W1A- Diffuse X-ray Scattering Beamline of 10.13039/501100014938Beijing Synchrotron Radiation Facility (https://cstr.cn/31109.02.BSRF.1W1A) for providing technical support and assistance in data collection.

## Author contributions

Conceptualization, J.L. and M.H.Y.; methodology, M.Y.M., G.Y. and J.Z.; investigation, X.Y.X., L.L.Z., M.Y.M., G.Y., and J.Z.; visualization, X.Y.X.; writing – original draft, X.Y.X., L.L.Z. and J.L.; writing – review and editing, X.Y.X., L.L.Z. and J.L.; supervision, M.H.Y. and J.L.

## Declaration of interests

The authors declare no competing financial interest.

## STAR★Methods

### Key resources table

**Table:** 

REAGENT or RESOURCE	SOURCE	IDENTIFIER
**Chemicals, peptides, and recombinant proteins**		

Conjugated polymer DPP5O	Synthesized according to literature methods	Ma et al.^3^
Conjugated polymer Pg2T-TT	Synthesized according to literature methods	Giovannitti et al.^52^
Photo-crosslinker DtFDA	Synthesized according to literature methods	Lai et al.^44^
Photoresist AZ 5214 and developer NMD-3	Commercially purchased	AZ Electronic Materials
Photoresist SU-8 and developer PFMEA	Commercially purchased	MicroChem, SU8 1040
Solvents (chloroform, hexafluoroisopropanol, acetone, isopropanol)	Commercially purchased	https://www.energy-chemical.com/

**Software and algorithms**		

Zahner Analysis (for EIS data fitting)	Zahner	http://www.zahner.com.cn/
Origin 2023	OriginLab	https://www.originlab.com/

### Experimental model and study participant details

There are no experimental models (animals, human subjects, plants, microbe strains, cell lines, primary cell cultures) used in the study.

### Method details

#### Materials

The conjugated polymers (DPP-5O and Pg2T-TT) and photo-crosslinkable small molecule (DtFDA) were synthesized following previously reported procedures.^3^^,^^44^^,^^52^ Figure 1 shows the chemical structures of DPP-5O, Pg2T-TT, and DtFDA. All other chemicals were purchased from commercial suppliers and used without further purification.

#### Device fabrication

Organic electrochemical transistors (OECTs) were fabricated on glass substrates to evaluate the electrical performance of semiconductor polymer blends with the crosslinker, DtFDA. The glass substrates were sonicated for 20 minutes each in an aqueous detergent solution, deionized water, acetone, and isopropanol, and then dried by blowing nitrogen. After treating the cleaned substrates with a VP-RS6 UV ozone cleaner for 420 seconds, the photoresist (AZ 5214) was spin-coated at 1500 rpm for 40 seconds and then baked at 150°C for 2 minutes in air to remove residual solvents. Using an MDA-400M mask aligner, the photoresist was exposed to UV light for 10 seconds and developed with NMD-3 developer for 60 seconds. Source and drain electrodes were defined by thermally evaporating 5 nm of Cr and 40 nm of Au under a vacuum of 10^-4^ Pa. The metal was lifted off in acetone, then rinsed with isopropanol. The geometry factors (W/L) of the yielded OECT devices were 1600 μm/20 μm, 3200 μm/20 μm, 4800 μm/20 μm, and 1600 μm/30 μm.

For depositing the active layer of OECTs, blends of the crosslinker DtFDA (10 mg/mL in chloroform) and the n-type polymer semiconductor DPP-5O (5 mg/mL in chloroform) were prepared at weight ratios of 2:100 (2wt%), 5:100 (5wt%), 10:100 (10wt%), and 15:100 (15wt%). As shown in Figure 2, the polymer blends with DtFDA were spin-coated at 2000 rpm for 30 s and photo-crosslinked by exposure to 365 nm UV light through a mask for 10 minutes. Then the films were developed in chloroform for 10 s and dried under a nitrogen stream.

The inverter circuits were fabricated using the above photolithography and lift-off process. To integrate the p-type and n-type active layers on a cofacial inverter, Pg2T-TT and DPP-5O were blended with DtFDA at a weight ratio of 100:10, respectively, and then photo-patterned on the inverter channels with distinct pattern edges (Figure S1). SU-8 was patterned according to standard procedures to serve as the encapsulation layer.

For electrochemical impedance spectroscopy (EIS) measurements, 5 nm of Cr and 40 nm of Au were thermally evaporated through a square metal mask onto cleaned glass slides. Then SU-8 layers were employed to define various active areas (2, 4, 6, and 9 mm^2^) and serve as the encapsulation layer. Polymer films were prepared using the same coating process, and their thickness was measured with a KLA Tencor P7 profilometer.

#### Characterization

##### Characterization of optical profilometer

The surface morphology and patterning fidelity of the semiconductor thin films were characterized using an optical profilometer (Bruker 3D Optical Profilometer Contour GT-I). The profilometric analysis demonstrated that the patterned semiconductor structures on silicon substrates exhibited excellent edge definition, uniform thickness distribution, and sub-micrometer pattern fidelity, confirming the effectiveness of the fabrication process.

##### UV-vis-NIR absorption spectroscopy and spectroelectrochemistry

UV-vis-NIR absorption spectroscopy and Spectroelectrochemistry were characterized with a Shimadzu UV 3700 UV-vis spectrometer. Blends of DtFDA and DPP-5O were prepared at weight ratios of 0 (pristine DPP-5O), 2:100 (2 wt%), 5:100 (5 wt%), 10:100 (10 wt%), and 15:100 (15 wt%) in dilute chloroform solution (10^-5^ mol/L). Spectroelectrochemistry was performed using an ITO-coated glass slide coated with polymer films. The samples were spin-coated with DPP-5O and blends of DtFDA and DPP-5O (10 wt%) at 1000 rpm for 30 s, then exposed to 365 nm UV light for 10 minutes to crosslink the thin films. These polymer-coated ITO slides were used as the working electrode (WE) and immersed in a cuvette containing 100 mM NaCl (aq). A Pt wire was used as the counter electrode (CE), and an Ag/AgCl electrode served as the reference (RE). A Shimadzu UV 3700 UV-vis spectrometer was used, with the beam path passing through the cuvette and the ITO samples. A background spectrum was recorded with the cuvette/electrolyte/ITO setup before a doping potential was applied to the cell. Each doping potential was applied to the WE for 30 s before recording the spectra (to achieve a stable doping level) and was maintained for a specified period until the completion of spectrum scanning.

##### Electrochemical impedance spectroscopy

Electrochemical impedance spectroscopy (EIS) was performed by Zahner Zennium XC with 100 mM NaCl (aq) as the electrolyte. The Au electrode and the polymer films served as working electrodes. The Ag/AgCl electrode was employed as the reference electrode. The platinum wire served as the counter electrode. In EIS measurements, the potential was set equal to V_G_ (when g_m_ achieves a maximum value). The scanning frequency was from 100 mHz to 100 kHz, and the AC amplitude of the potential in the form of a sine wave was set at 10 mV. The volumetric capacitance (C∗) was calculated by the formula $C^{*}=\frac{C_{\text{eff}}}{V}$, where V is the volume of polymer film, and C_eff_ could be calculated by the relation $C_{\text{eff}}=C_{\text{eq}}{\left(2\pi \right)}^{\alpha -1}\;\sin\left(-\frac{\pi \alpha }{2}\right)$. Zahner Analysis was used to extract the C_eq_ and α by fitting the bond rheogram with Equivalent Circuit: R_0_+R_1_/CPE+R_2_/C, where R_0_, R_1_, R_2_ are the electrolyte resistance, polymer resistance, and resistance to calibrate fitting error, respectively. CPE and C are the constant phase element and capacitance elements in the polymer film, respectively, to calibrate the fitting error. The film thickness was measured in a dry state using a KLA Tencor P7 profilometer.

##### Grazing-incidence wide-angle X-ray scattering

Grazing-incidence wide-angle X-ray scattering (GIWAXS). The GIWAXS data were obtained at 1W1A- Diffuse X-ray Scattering Beamline of Beijing Synchrotron Radiation Facility (BSRF-1W1A). The incidence angle was 0.2°.

##### OECT and inverter measurements

The fabricated transistors were characterized using a Keithley 2902B in an aqueous solution under ambient air. The following Bernards’ model calculated the OECT performance parameters of the polymers:

$$g_{m}=\frac{\text{Wd}}{L}\mu C^{*}\left|\left(V_{\text{Th}}-V_{G}\right)\right|$$

where W is the channel width, d is the thickness of the polymer film, L is the channel length, *μ* is the charge carrier mobility, C∗ is the volumetric capacitance, V_Th_ is the threshold voltage, and V_G_ is the gate voltage. The figure-of-merit *μ*C∗ is the product of charge carrier mobility *μ* and volumetric capacitance C∗. During the tests, the polymer-coated channel was confined by a PDMS tank filled with 0.1 M NaCl (aq), and an Ag/AgCl pellet electrode was immersed in the electrolyte to serve as the gate electrode. For the p-type OECT measurements, the output curves were collected by varying V_D_ from 0 to −0.6 V and applying successive V_G_ steps from 0 to −0.8 V (0.1 V per step). Transfer curves were collected using a constant V_D_ = −0.6 V, while varying V_G_ from 0 to −0.7 V (sweeping speed is 40 mV/s). For the n-type measurements, the output curves were collected using a V_D_ from 0 to 0.4 V and a V_G_ from 0 to 0.7 V, and transfer curves were collected using a constant V_D_ = 0.4 V, while varying V_G_ from 0 to 0.8 V. The threshold voltage (V_Th_) of the p-type and n-type OECTs was extracted from a linear extrapolation of the |I_D_|^1/2^ versus V_G_ plot.

The VTCs of the inverter were collected using two Keithley 2902Bs to apply the input voltage (V_in_) and the working voltage (V_DD_), and to measure the output voltage (V_out_). The VTCs were collected by sweeping V_in_ from 0 to 0.6 V while holding V_DD_ constant at 0.3, 0.4, 0.5, and 0.6 V. Voltage gain was calculated by taking the derivative of the VTC (∂V_out_/∂V_in_).

### Quantification and statistical analysis

All data analysis was performed using OriginLab software (Origin 2023). Detailed statistical parameters for each experiment are reported in corresponding figure legends and/or results section of the main text.

### Additional resources

This study has not generated or contributed to a new website/forum or has not been part of a clinical trial.
