# Supplementary material for: Photopatterning of organic mixed ionic electronic conductors for monolithic complementary inverter
Source: iScience. 2026 Feb 9;29(3):114965. doi: 10.1016/j.isci.2026.114965 (PMC13080481; doi:10.1016/j.isci.2026.114965)
Supplement: Document S1. Figures S1–S10 and Table S1 [file mmc1.pdf]

iScience, Volume *29*

Supplemental information

Photopatterning of organic mixed ionic electronic conductors for monolithic complementary inverter

Xinyao Xie, Linlong Zhang, Mingyu Ma, Gang Ye, Jun Zhang, Xingxing Chen, Minghui You, and Jian Liu

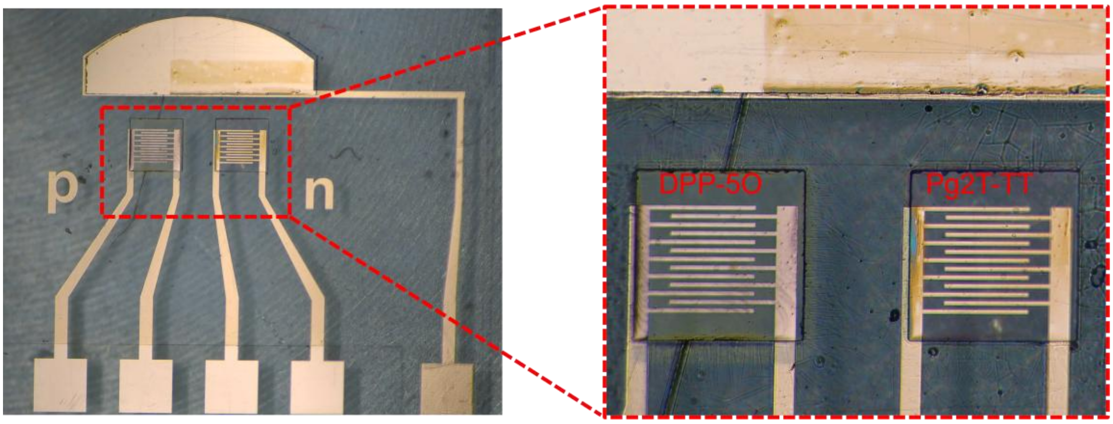

Figure S1: Optical image of cofacial complementary inverter device with patterned OMIECs.

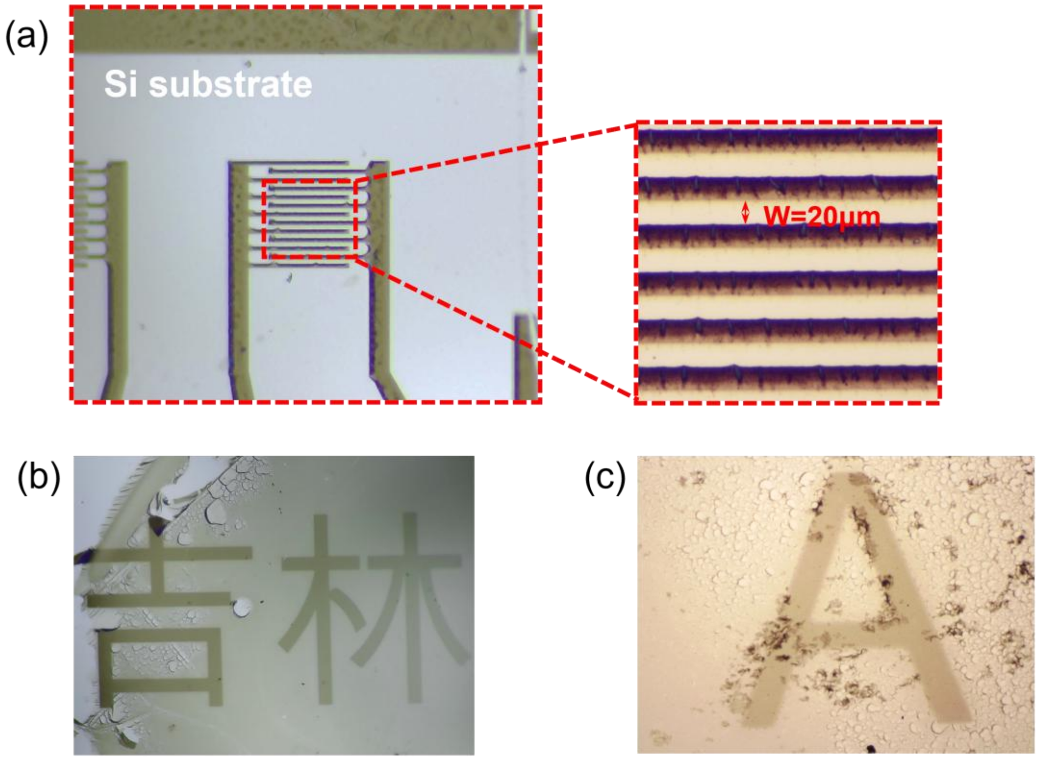

Figure S2: Optical images of patterned films. (a) Images of the patterned thin films of DPP-5O: DtFDA process with CF. (b) and (c) The thin films processed with HFIP.

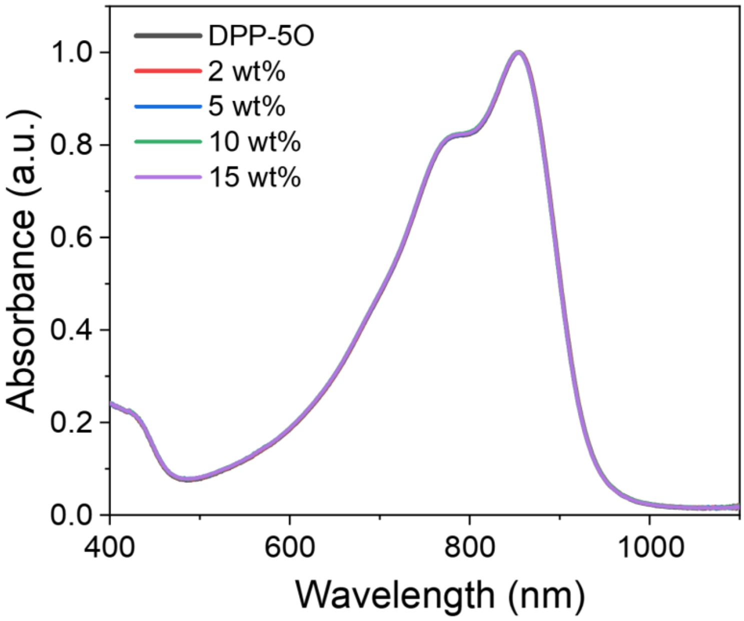

Figure S3: UV-vis-NIR absorption spectra of dilute solution blends of DtFDA and DPP-5O with different weight ratio of 0 (pristine DPP-5O), 2, 5, 10, 15 wt%, respectively.

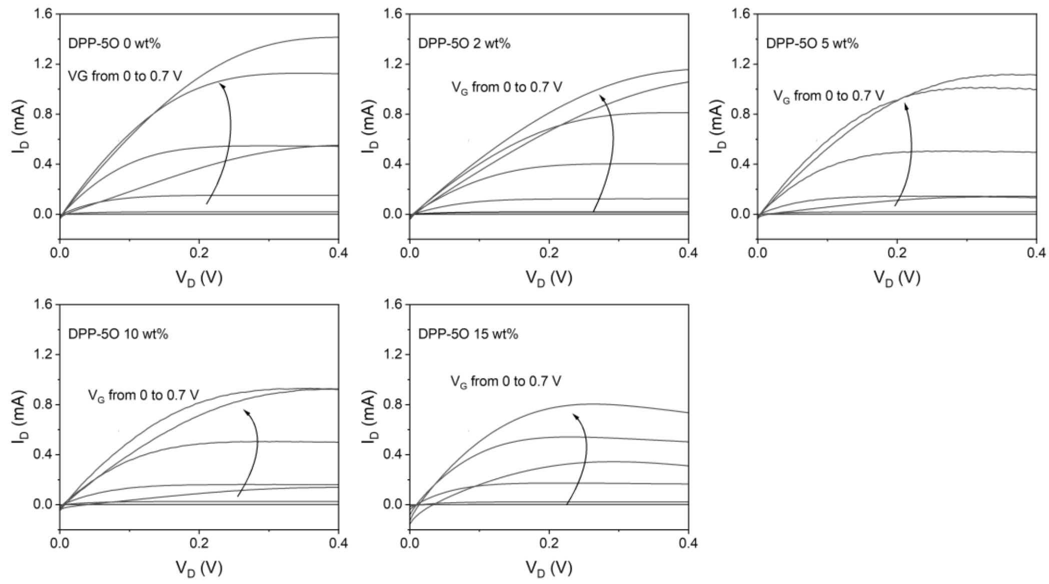

Figure S4: Output curves of OECT based on blends of DtFDA and DPP-5O. The weight ratios of DtFDA are 0 (pristine DPP-5O), 2, 5, 10, 15 wt%.

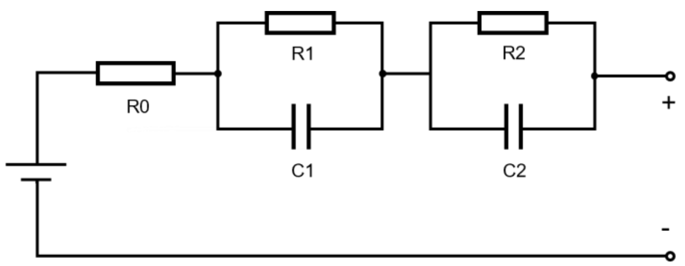

Figure S5: The equivalent circuit for analyzing electrochemical impedance spectroscopy (EIS) data.

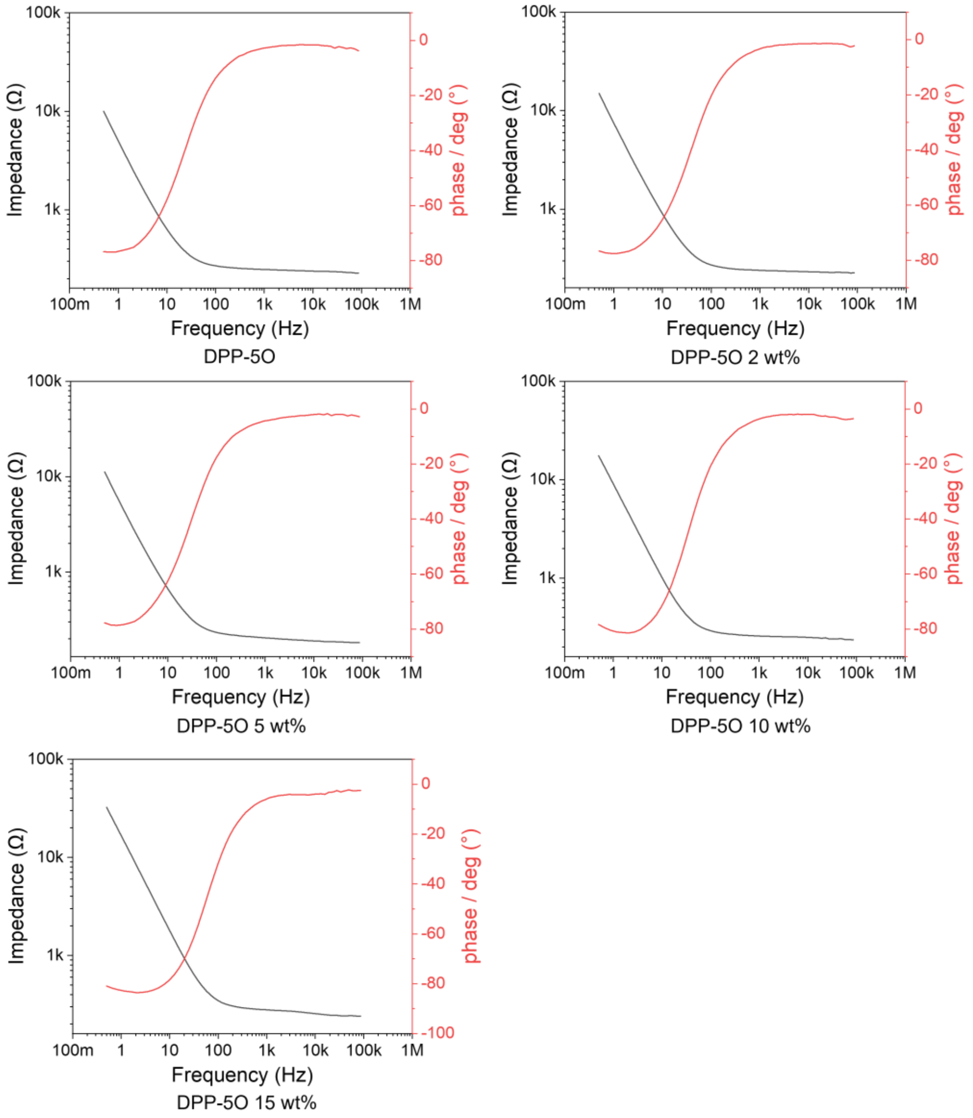

Figure S6: Electrical impedance spectroscopy of DPP-5O with different weight ratios of crosslinkers. The weight ratios of DtFDA are 0 (pristine DPP-5O), 2, 5, 10, 15 wt%.

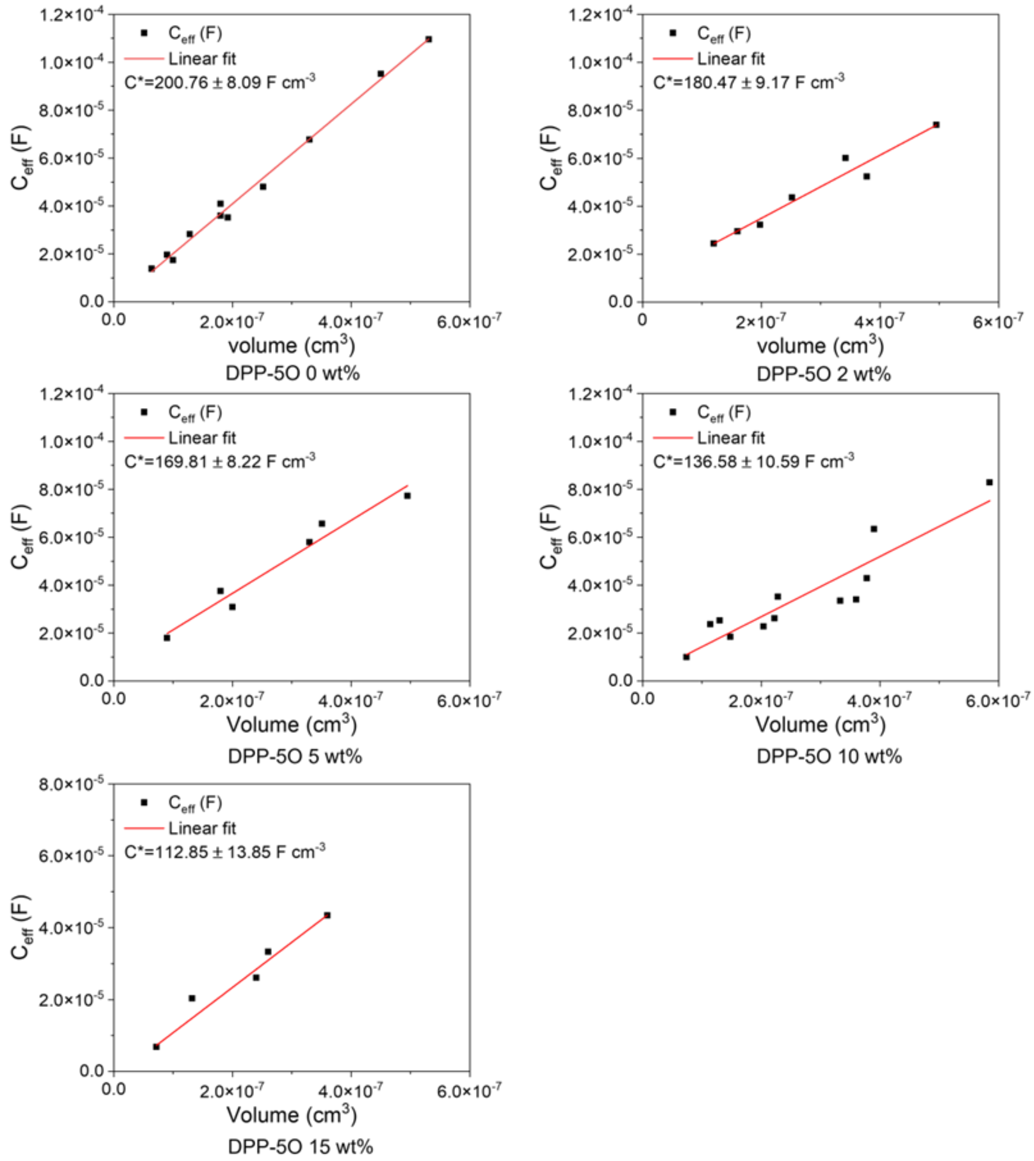

Figure S7: Volumetric capacitance of DPP-5O with different weight ratios of crosslinkers. The weight ratios of DtFDA are 0 (pristine DPP-5O), 2, 5, 10, 15 wt%.

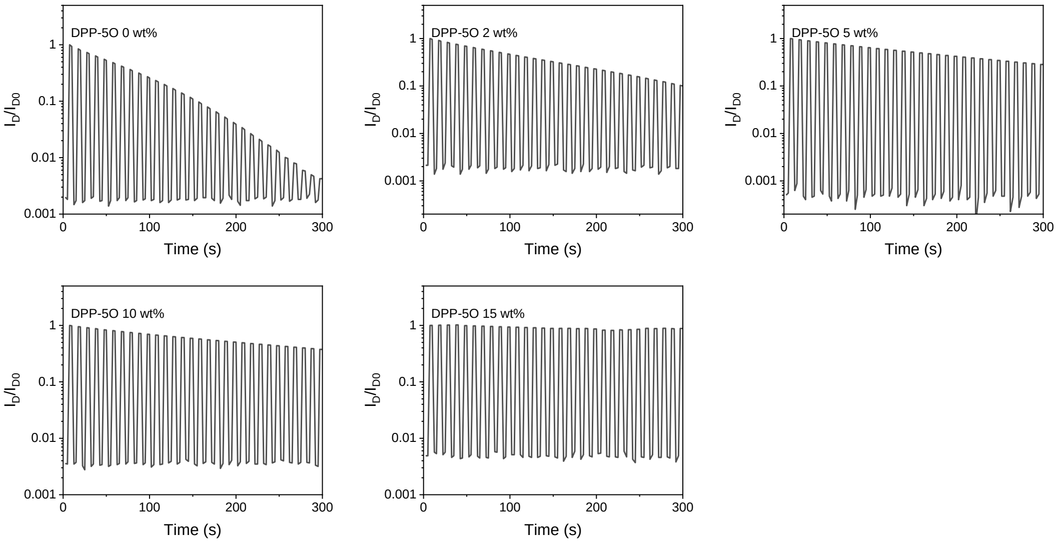

Figure S8: Operational cycling stability of DPP-5O with different weight ratios of crosslinkers, electrolyte: 0.1 M NaCl (aq). The weight ratios of DtFDA are 0 (pristine DPP-5O), 2, 5, 10, 15 wt%.

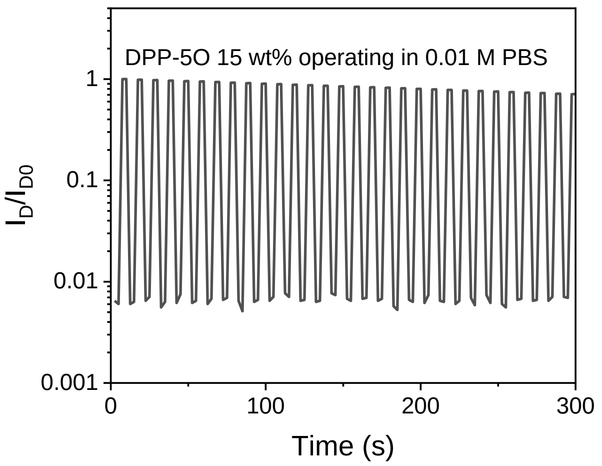

Figure S9: Operational cycling stability of DPP-5O with crosslinker weight ratio of 15%, electrolyte: 0.01 M PBS (aq).

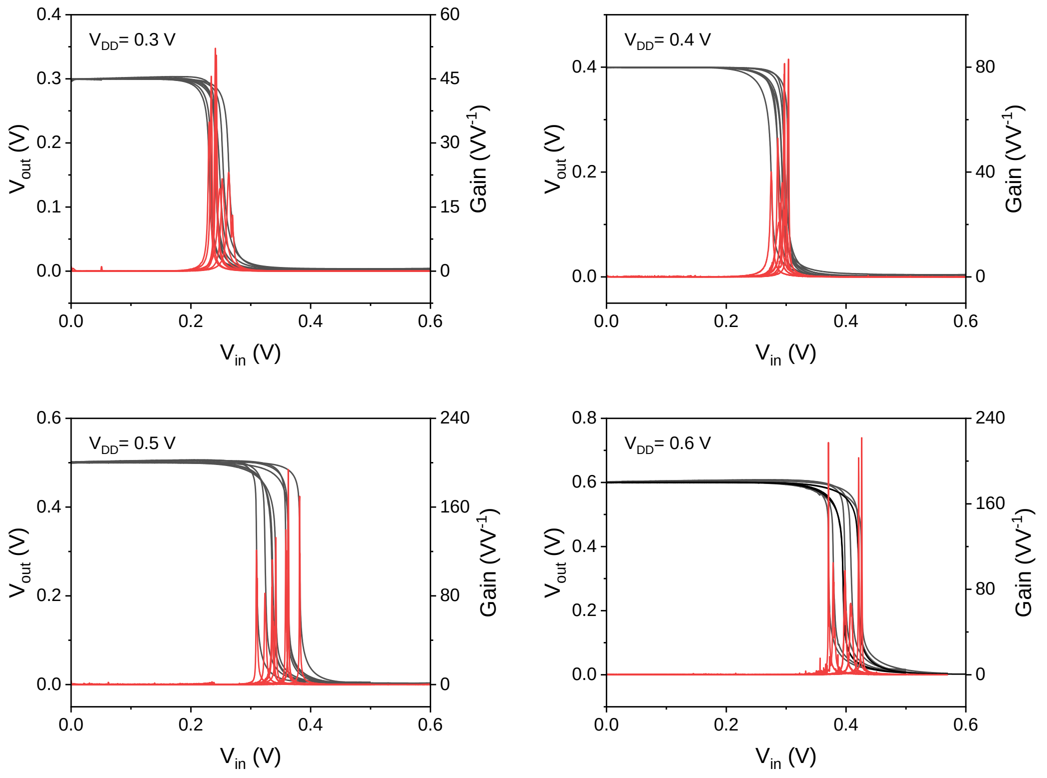

Figure S10: Voltage transfer characteristic curves and corresponding calculated voltage gains of the cofacial complementary inverter (n = 8). The VTC curves are measured under V_{DD}= 0.3, 0.4, 0.5, and 0.6 V, respectively.

Technique	Typical Resolution and Throughput	Impact on OMIEC Performance	Compatibility with Multilayer Integration	Limitations for OMIEC Circuits
This work: Photo-crosslinking Patterning	1-20 µm, high throughput	Significantly enhanced	Excellent	Resolution limited by optical diffraction
Conventional Photolithography[1, 2]	<1 µm, high	Typically severely degraded	Poor	Harsh chemicals incompatible with OMIECs
E-beam Lithography[3, 4, 5]	<10 nm, very low	Typically degraded	Poor	Expensive, slow and requires vacuum
Nanoimprint Lithography[6, 7]	<100 nm, medium	Degraded due to high pressure or temperature during imprinting	Medium	Template cost, contact process may damage soft films
Inkjet Printing[8, 9, 10]	10-50 µm, medium	Depends on ink formulation	Good	Limited resolution, challenges in film uniformity

Table S1: Comparison of high-resolution patterning techniques for organic mixed ionic-electronic conductors (OMIECs).

Reference

1. D. Cho, J. Park, J. Kim, T. Kim, J. Kim, I. Park, S. Jeon, Three-Dimensional Continuous Conductive Nanostructure for Highly Sensitive and Stretchable Strain Sensor. ACS Appl. Mater. Interfaces **9**, 17369-17378 (2017).

2. S. L. Xiang, Q.-C. Ding, J. Liu, H. Hu, B. Zhao, S. Z. Lu, F. Huang, J. Cunha, J. Rodrigues, Z. Yu, H. Yin, Photoresist systems in floating T-gate fabrication for GaN high electron mobility transistors. Nano Mater. Sci., in press (2024).

3. A. Xomalis, C. Hain, A. Groetsch, F. F. Klimashin, T. Nelis, J. Michler, J. Schwiedrzik, Resist-Free E-beam Lithography for Patterning Nanoscale Thick Films on Flexible Substrates. ACS Appl. Nano Mater. **6**, 3388-3394 (2023).

4. C. X. Zhu, H. Ekinci, A. X. Pan, B. Cui, X. L. Zhu, Electron beam lithography on nonplanar and irregular surfaces. Microsyst. Nanoeng. **10**, 52 (2024).

5. C. F. de Lima, P. Thamizhavel, T. J. Woehl, Electron-Matter Interactions During Electron Beam Nanopatterning. Adv. Funct. Mater., e21859 (2025).

6. Z. W. Yang, H. T. Zhao, X. G. Qi, C. Y. Yao, R. Ma, J. S. Song, Y. X. Cao, D. Y. Ma, H. M. Li, G. X. Cui, J. Zhang, Novel ultraflexible transparent self-heating composite films for nanoimprint lithography. Mater. Des. **254**, 114123 (2025).

7. P. H. Wang, C. M. Wang, Review of nanoimprinted photonics. Nanotechnology **36**, 442002 (2025).

8. K. Liang, Y. Wang, S. S. Shao, M. M. Luo, V. Pecunia, L. Shao, J. W. Zhao, Z. Chen, L. X. Mo, Z. Cui, High-performance metal-oxide thin-film transistors based on inkjet-printed self-confined bilayer heterojunction channels. J. Mater. Chem. C **7**, 6169-6177 (2019).

9. T. Pandhi, A. Chandnani, H. Subbaraman, D. Estrada, A Review of Inkjet Printed Graphene and Carbon Nanotubes Based Gas Sensors. Sensors **20**, 5642 (2020).

10. T. L. Cao, Z. J. Yang, H. Zhang, Y. M. Wang, Inkjet printing quality improvement research progress: A review. Heliyon **10**, e30163 (2024).
